# Proteomic Characterization of Lytic Bacteriophages of *Staphylococcus aureus* Isolated from Sewage Affluent of India

**DOI:** 10.1155/2014/265298

**Published:** 2014-09-14

**Authors:** Kamalpreet Kaur Sangha, B. V. Sunil Kumar, Ravi Kant Agrawal, Dipak Deka, Ramneek Verma

**Affiliations:** ^1^School of Animal Biotechnology, Guru Angad Dev Veterinary and Animal Sciences University, Ludhiana, Punjab 141004, India; ^2^Indian Veterinary Research Institute, Izatnagar, Bareilly, Uttar Pradesh 243122, India

## Abstract

*Staphylococcus aureus* is a Gram-positive bacterium that causes a variety of diseases, including bovine mastitis, which has severe economic consequences. Standard antibiotic treatment results in selection of resistant strains, leading to need for an alternative treatment such as bacteriophage therapy. Present study describes isolation and characterization of a staphylococcal phage from sewage samples. *S. aureus* isolates obtained from microbial type culture collection (MTCC), Chandigarh, India, were used to screen staphylococcal phages. A phage designated as ΦMSP was isolated from sewage samples by soft agar overlay method. It produced clear plaques on tryptone soya agar overlaid with *S. aureus*. Transmission electron microscopy revealed that the phage had an icosahedral symmetry. It had 5 major proteins and possessed a peptidoglycan hydrolase corresponding to 70 kDa. ΦMSP infection induced 26 proteins to be uniquely expressed in *S. aureus*. This phage can be proposed as a candidate phage to treat staphylococcal infections.

## 1. Introduction

*Staphylococcus aureus* (*S. aureus*) is a nonmotile and nonspore forming Gram-positive bacterium responsible for a number of diseases in animals and human beings. It is a common cause of nosocomial pneumonia and a variety of diseases including food poisoning, toxic shock syndrome, skin related diseases, and bovine mastitis; the latter has severe economic consequences.* S. aureus* has acquired resistance to most of the antibiotics, from the oldest (penicillin) to the latest (linezolid) [1]. The resistance of bacterial pathogens to most of the available antibiotics has become a grave medical and veterinary problem all over the world. No new class of antibiotics has been discovered in the last 40 years and, therefore, there is a pressing need for the development of some alternatives to antibacterial therapy to combat multiple drug resistant bacterial infections.

Bacteriophages were first used successfully to treat bacterial infections a decade before antibiotics were discovered [2]. To compensate for the shortcomings of chemotherapy, bacteriophage therapy has been developed against many bacterial infections [2, 3]. Phages are now used in many applications of biotechnology and in medical fields as alternatives to antibiotics, vector for protein, and DNA vaccines or as gene therapy delivery vehicles. Potential applications of the phage therapy include treatment and prevention of purulent wound infections, in pre- and postoperative surgery, burn wounds, skin infections, eye and ear infections [4], and diseases of the lungs and pleura [5]. All these observations suggest that bacteriophages can successfully be used to combat antibiotic resistance. In this paper, we describe isolation of a lytic bacteriophage against* S. aureus* and its characterization.

## 2. Materials and Methods

### 2.1. Collection of Bacterial Strains and Their Maintenance

*S. aureus* was procured from microbial type culture collection and gene bank (MTCC), Chandigarh. The freeze-dried culture was suspended in 3% tryptone soya broth (TSB) and plated onto tryptone soya broth (HiMedia, Mumbai) supplemented with 1.5% bacteriological agar (HiMedia, Mumbai). The plates were incubated at 37°C overnight. The following day, colonies were picked up from the plates and cultured in TSB. The cultures were further confirmed by Gram's staining [6].

### 2.2. Isolation of Phages against* S. aureus*

Lytic phages against* S. aureus* were screened by soft agar overlay method [7] following an enrichment procedure. About 30 samples were collected from sewages of Guru Angad Dev Veterinary and Animal Sciences University (GADVASU), Ludhiana. The samples were centrifuged at 6000 rpm for 10 minutes. The supernatant was collected and filtered through 0.45 *μ* syringe filter. About 150 *μ*L of chloroform was added to 15 mL of the filtrate and incubated for 20 minutes. The filtrate was mixed with 10 mL* S. aureus* culture (in log phase) and 25 mL of 2X tryptone soya broth (6%) and incubated at 37°C overnight. The following day the above mixture was centrifuged and filtered through 0.22 *μ* syringe filter. A 100 *μ*L of bacterial culture (in log phase) and 50 *μ*L of purified phage filtrate were incubated for 20 minutes and added to 3 mL of molten soft agar, spread on tryptone soya agar plates and incubated at 37°C for 18 hours. After incubation, the formation of cleared zones (plaques) suggested the presence of lytic phages. The plaques were picked, resuspended in salt of magnesium (SM) buffer (100 mM NaCl, 8 mM MgSO_4_, 50 mM Tris-HCl (pH 7.5), and 0.01% gelatin), and incubated with the host to obtain lysis plaques. After primary isolation, secondary streaking was done with the plaques from positive primary plates on TSA plates with host bacteria. The plates were incubated at 37°C for 15–18 hours to obtain lines of clearances [7].

### 2.3. Elution of Phages

SM buffer was poured on the phage grown secondary plates. The entire surface of semisolid material was scrapped with sterile tips and plates were incubated at 37°C for 8 hours. The following day the entire scrapping material with SM buffer was collected with wide bore microtips into 50 mL centrifuge tubes. The phage suspension was centrifuged at 6000 rpm for 5 minutes at 4°C. The supernatant was filtered through 0.22 *μ* syringe filter and stored at 4°C for further use.

PFU count of the isolated phages was determined as per Chandra et al. (2011) [8] using the formula PFU count = number of plaques counted × dilution/volume of phage preparation used (i.e., 100 *μ*L).

### 2.4. Concentration of Phage Filtrate by Ultracentrifugation

The phage filtrate was further concentrated by ultracentrifugation as per Eyer et al. (2007) [9] with slight modifications. Briefly, 10 mL of the phage filtrate was subjected to ultracentrifugation at 2,50,000 g for 2.5 hours at 4°C. The pellet obtained was then reconstituted in 100 *μ*L of SM buffer and stored at 4°C. PFU of the concentrated phage was determined again following the same protocol as mentioned earlier.

### 2.5. Transmission Electron Microscope (TEM) Imaging of Phages

Morphological characterization of the phage was done by electron microscopy, which was performed at All India Institute of Medical Sciences, New Delhi. A 6 *μ*L of a concentrated phage suspension (10^10^ PFU/mL) in SM buffer was spotted on top of a hydrophilic Formvar carbon-coated copper grid (Nissin EM Corporation) and the sample was allowed to adsorb for 2 minutes. Excess sample was removed carefully by touching the side of a grid with filter paper; then 6 *μ*L of deionized water was spotted on the grid and removed after a short time. Phage was stained by addition of 1% aqueous phosphotungstic acid (PTA), pH 6.5. After 2 minutes, excess stain was removed, and the grid was allowed to air-dry for 30 minutes. The grids were observed under transmission electron microscope (Morgagni 268 D, Fei Electron Optics) [10].

### 2.6. Bacteriophage Proteome Analysis

#### 2.6.1. Detection of Proteins by SDS-PAGE

Proteins of the isolated* Staphylococcus* phage were detected by sodium dodecyl sulfate-polyacrylamide gel electrophoresis [11]. Concentrated phage preparation (10^10^ PFU/mL approx.) was mixed with equal volume of 2X Laemmli buffer (Sigma, USA) and boiled for 10 minutes. A 25 *μ*L sample mixture was loaded in each well along with 6 *μ*L of broad range prestained protein ladder** (**Puregene, USA**)** in one well. The gel was run at 20 mA of constant current for 1 hour (approx.) till the tracking dye reached the bottom of the gel. The gel was finally stained with 0.25% Coomassie brilliant blue R-250 stain (Amresco, USA) for 4 hours and then suitably destained for best visibility of the protein bands with several changes of destaining solution and stored in 7% glacial acetic acid solution for photography. The gel was scanned in a scanner and documented.

#### 2.6.2. Infection of* Staphylococcus aureus* by Its Bacteriophage

*S. aureus* grown overnight was inoculated into two 20 mL fresh TSB tubes and incubated at 37°C for 6 hours. One of the tubes was infected with 1 mL of isolated phage (10^10^ PFU/mL) while the other was kept as control. Both tubes were incubated at 37°C for the next 8 hours. The bacteriophage infected and noninfected* S. aureus* cells were harvested by centrifugation at 8000 rpm for 10 minutes. After three washes with sterile phosphate-buffered saline (PBS) (pH 7.4), the cells were lysed by incubating at room temperature for 1 hour with lysis buffer (8 M urea, 4% w/v 3-cholamidopropyl-dimethylamminio-1-propane sulphonate (CHAPS), 2% immobiline pH gradient (IPG) buffer (3–10 pH range), 10 mM dithiothreitol (DTT), and cocktail protease inhibitor (Fisher Scientific, USA)). The mixture was then sonicated with microtip (Misonix, USA) for 5 brief (20–30 sec) cycles of amplitude 15 Hz with 30 seconds cooling on ice in between each sonication. The suspension was centrifuged at 12000 rpm for 8 minutes and the supernatant was collected. Protein concentration was determined using NanoDrop spectrophotometer (Thermo Scientific, USA). Whole cell protein was purified using 2DE-clean up kit (GE Healthcare, Sweden). Protein pellet was reconstituted in rehydration buffer (8 M urea, 2% CHAPS, 0.2% DTT (dithiothreitol), 0.5% IPG buffer pH 3–10, and 0.002% bromophenol blue) and the concentration was determined.

### 2.7. 2-Dimensional Electrophoresis (2DE) and Image Analysis

The first-dimension electrophoresis of the protein sample (about 200 *μ*g) was conducted on immobilized pH gradient gel (IPG) strips (pH 3 to 10; 7 cm; GE Healthcare, USA). The IPG strip was rehydrated at room temperature for 12 hours and subjected to isoelectric focusing using a 5-step program (step/hold 100 V for 4 hours, s/h 500 V for 1 hour, gradient 1000 V for 1 hour, gradient 5000 V for 2 hours, s/h 5000 V for 30 minutes, and maximum current of 50 *μ*A/IPG strip). Subsequently, the IPG strips were incubated in equilibration buffer I (6 M urea, 50 mM Tris-HCl, 2% SDS, 30% glycerol, 0.002% bromophenol blue, pH 8.8) containing 1% (wt/vol) DTT followed by equilibration buffer II (equilibration buffer I + 2.5% iodoacetamide) for 15 minutes in each. The second-dimension separation was carried out on 12% SDS-PAGE at 100 V for 30 minutes and then 200 V until the dye front reached the bottoms of the gels. After electrophoresis, the gels were stained with Coomassie blue R-250 and scanned using an Imagescanner III (GE Healthcare). Spot detection, spot matching, and quantification analysis were performed using ImageMaster 7.0 2D analysis software (GE Healthcare). 2D analysis was biologically repeated three times. The gel images were normalized according to the total quantity in the analysis set. Student's *t*-test was adopted to evaluate the spots that were significantly different between phage infected and noninfected* S. aureus* groups.

### 2.8. Zymogram Analysis

Purified phage suspension was dialyzed against SM buffer, mixed with loading buffer (1% SDS, 6% sucrose, 100 mM dithiothreitol, 10 mM Tris, pH 6.8, and 0.0625% bromophenol blue), and boiled for 5 minutes before loading onto 12% SDS-PAGE gels containing 0.2%* S. aureus* autoclaved cells [12]. Gels were cast according to Laemmli (1970) [11], except that only 0.01% SDS was used to allow protein renaturation. A prestained protein ladder was also loaded in one well (Puregene, USA). After electrophoresis, the lane with prestained protein ladder was cut and separated from the rest of the gel for comparison after staining. The gel was washed for 30 minutes with water and then soaked for 1 day at room temperature in 150 mM sodium phosphate buffer, pH 7.0, containing 0.1% Triton X-100 and 10 mM MgCl_2_. Zymogram was stained for 3 hours with 0.1% methylene blue in 0.001% KOH and washed with water.

## 3. Results

### 3.1. Isolation of Lytic Phages against* Staphylococcus aureus*

**Figure 1:**
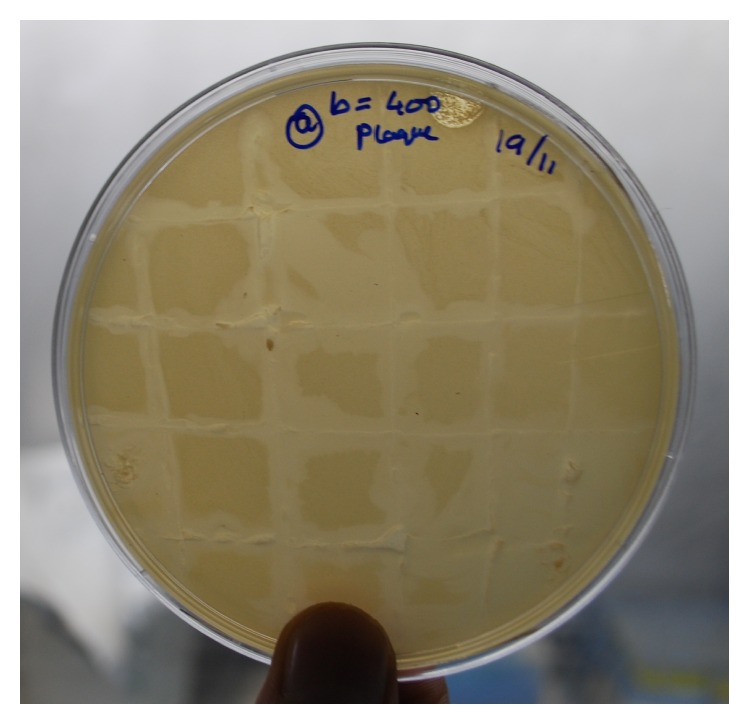
Secondary streaking from the plaques of ΦMSP on a lawn of* S. aureus* showing lines of clearance.

Screening of different sewage samples of GADVASU, Ludhiana, by soft agar overlay method yielded a lytic phage against* S. aureus* designated as ΦMSP. It formed very small zones of clearances (plaques) on the lawn of* S. aureus*. These plaques were further used for secondary streaking on 1.5% TSA plates overlaid with* S. aureus*. Zones of clearances were observed across the lines of streaking (Figure 1). This indicated that the phages isolated had potent lytic activity against* S. aureus*. Phage titre (plaque forming units)/mL of ΦMSP was found to be 150 × 10^4^ PFU/mL.

### 3.2. Morphological Characterization of ΦMSP

**Figure 2:**
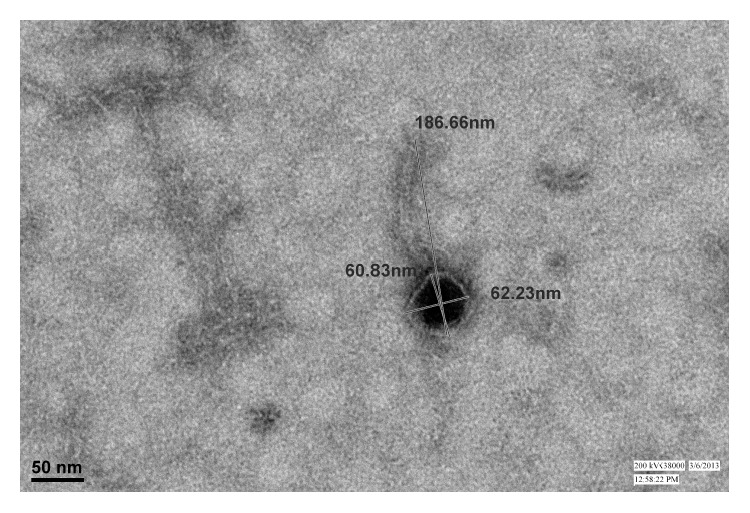
TEM images of bacteriophage ΦMSP. Bar, 100 nm.

Transmission electron microscopy of ΦMSP showed that the phage had an icosahedral head, about 60 nm in diameter, and a long noncontractile tail about 125.83 nm (Figure 2).

### 3.3. Proteomic Characterization

**Figure 3:**
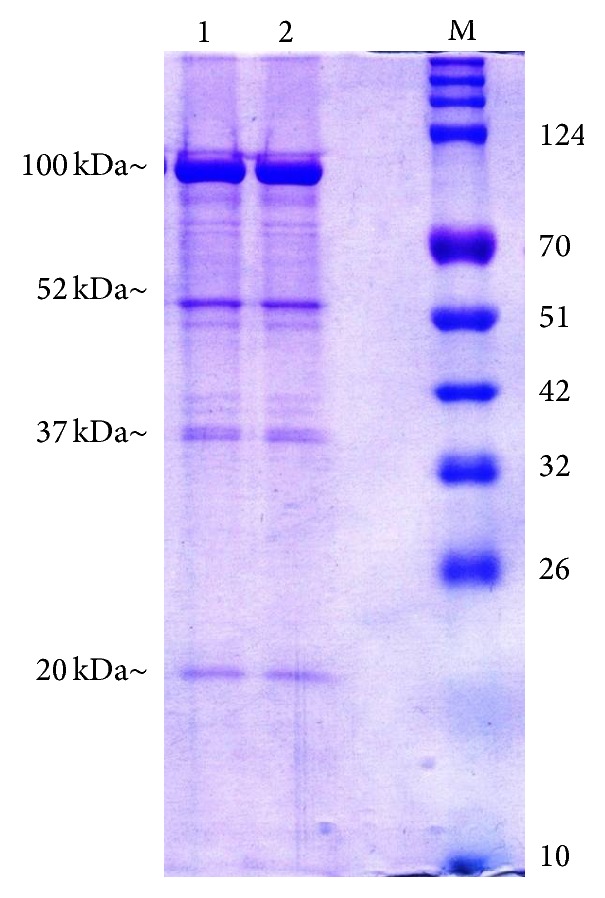
SDS-PAGE analysis of phage proteins (M: protein ladder; 1, 2: ΦMSP proteins).

Due to the relative simplicity of phage proteomes, 1DE was selected for initial proteomic analysis of the isolated* Staphylococcus* phage. Approximately 17 bands of proteins in 15–120 kDa range were detected (Figure 3). Out of these, 5 were found to be major proteins corresponding to molecular weights of 100 kDa, 52 kDa, 37 kDa, 35 kDa, and 15 kDa (approx.).

**Figure 4:**
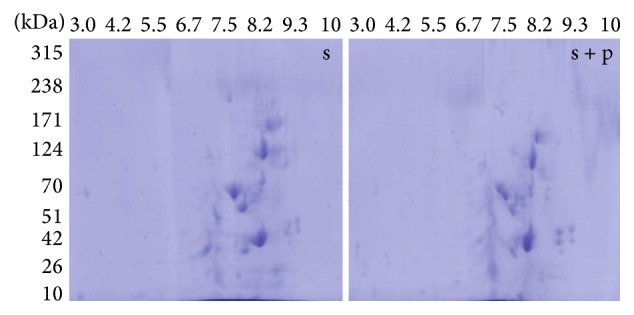
2DE gels showing proteomes of* Staphylococcus aureus* (s) and phage infected* Staphylococcus aureus* (s + p).

2DE was performed to identify proteins uniquely expressed in* S. aureus* in response to ΦMSP infection. This was done by comparing proteomes of* S. aureus* and phage infected* S. aureus* (Figure 4). A total of 123 and 149 protein spots were detected for* S. aureus* and phage infected* S. aureus,* respectively, suggesting that about 26 proteins were expressed in the bacteria in response to phage infection. Few differentially expressed proteins of* S. aureus* in response to phage infection are enlisted in Tables 1(a) and 1(b) along with their respective molecular weights (MW) and isoelectric points (pI). About 16 proteins (MW 30–70 kDa, pI 4–8) were found to be overexpressed while 5 proteins (MW 33–98 kDa, pI 4.8–6.2) were found to be underexpressed in* S. aureus* due to phage infection.

**Figure 5:**
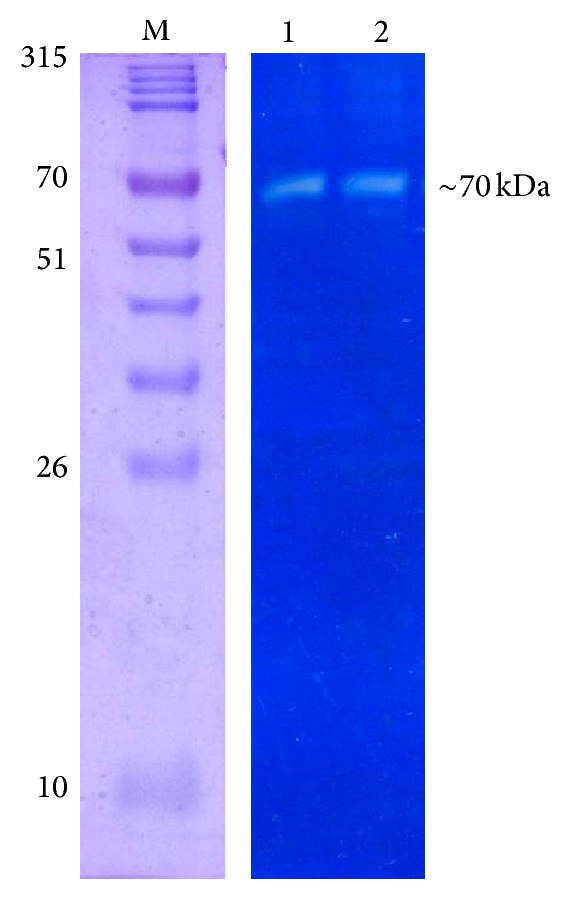
Zymogram gels of ΦMSP phages (M: prestained protein ladder (PureGene, USA); 1, 2: peptidoglycan hydrolase).

To confirm the presence of structural components with peptidoglycan hydrolytic activity in ΦMSP, zymogram assay was performed with autoclaved* S. aureus* cells. Upon electrophoretic separation of the phage proteins, the zymogram revealed a single 70 kDa band as a clear zone against a dark blue background of stained peptidoglycan (Figure 5).

## 4. Discussion

This paper describes isolation of a bacteriophage from sewage affluent which exhibited lytic capabilities with* S. aureus*. In the previous studies, workers had isolated phages from sewages with the ultimate aim of controlling* S. aureus* infections [13, 14]. In our effort to isolate novel staphylococcal phages, 30 sewage samples were screened and finally a lytic phage against* S. aureus* was isolated.

The isolated bacteriophage was selected for further characterization based on its capability of producing very clear lysis in plaque assay with* S. aureus*. A lytic (virulent) bacteriophage will enter a bacterial cell and use the cell's own machinery to multiply. Once these mechanisms are exhausted, the cell lyses and hundreds of copies of the original bacteriophage are released [15, 16]. Temperate bacteriophages do not always lyse the host cell and are capable of integrating their genome into the host genome, a process that can result in the horizontal transfer of genes, such as those associated with toxin production, virulence, and antibiotic resistance [17, 18]; they cannot be used in phage therapy. Lytic bacteriophages are preferred as targets for use in bacteriophage therapy [19].

TEM observation of the phage ΦMSP revealed that it had an icosahedral head/capsid and a noncontractile tail and was similar to the members belonging to Siphoviridae family according to the guidelines of the International Committee on Taxonomy of Viruses.

Essential to the analysis of virus-host interaction is the identification of the proteins/genes involved in virus infection. In the present study, based on the comparison of protein expression profiles of* S. aureus* and phage ΦMSP infected* S. aureus*, 26 staphylococcal proteins were revealed to be induced due to phage infection under laboratory conditions. The 26 staphylococcal proteins might have diverse metabolic functions. ΦMSP might either harness the host cell for its own reproduction by efficient takeover and reprogramming of the host physiology or replicate within the cell without affecting major biosynthetic pathways during the period of progeny production. The latter strategy might ensure phage propagation without the induction of host defense mechanisms, while the first strategy likely activates host responses. The induction of host defense systems would probably have an adverse effect on phage progeny production and decrease phage fitness [20]. It could be inferred that the direct stress response to virus infection might be ensured by regulating the expression of key proteins relevant to energy metabolism, transportation proteins and regulatory proteins of tricarboxylic acid cycle (TCA). Similar findings have been reported by Wei and Zhang (2010) [21], who have compared the proteomes of deep-sea thermophile* Geobacillus* sp. and bacteriophage GVE2 infected* Geobacillus* sp. and found that 20 proteins are involved in virus infection in the bacteria.

Mature virions are often endowed with peptidoglycan hydrolases involved in host cell wall degradation prior to injecting their genetic material during infection. Recently, it has been demonstrated that some tail tape measure proteins have structural domains similar to peptidoglycan hydrolases, enabling the entry of phage DNA through the thick peptidoglycan layer of host bacteria such as* Mycobacterium* [12]. In the present study, a single 70 kDa band in zymogram confirmed presence of active peptidoglycan hydrolase in each phage. The size is consistent with the virion protein gp58 (72.5 kDa) of phiIPLA88, which was isolated against* S. aureus* from mastitis milk and contained the predicted peptidoglycan hydrolytic domains [12].

## Figures and Tables

**(a):** 

S. number	Match count	MW	PI	*S. aureus *	*S. aureus *infected with phage	ANOVA
1.	2	37.1	8.0	0.104138	1.484754	0.106551
2.	2	46.8	6.5	2.11876	3.113429	0.016229
3.	2	29.3	4.3	1.504242	2.553671	0.828518
4.	2	43.9	5.0	2.25802	3.553182	0.214956
5.	2	65.1	6.0	1.543555	4.030283	0.258189
6.	2	69.2	7.9	0.10572	1.174222	0.135286
7.	2	41.2	5.2	0.23495	1.0649717	0.151667
8.	2	51.3	5.0	1.38565	2.47677	0.082358
9.	2	47.2	6.9	1.255836	2.513259	0.15945
10.	2	64.6	4.6	0.059875	1.9739796	0.0369
11.	2	35.1	5.9	0.145453	2.1237368	0.067126
12.	2	61.2	4.8	1.22356	2.866057	0.215685
13.	2	38.5	7.4	0.0025661	0.415909	0.333948
14.	2	49.2	5.6	0.189645	2.144631	0.373952
15.	2	25.6	7.2	0.019056	1.772107	0.087815
16.	2	71.2	3.9	0.0115645	1.918856	0.39799

**(b):** 

S. number	Match count	MW	PI	*S. aureus *	*S. aureus *infected with phage	ANOVA
1.	2	33.4	5.0	2.04945	1.365822	0.089914
2.	2	98.2	4.8	3.84341	1.150327	0.377635
3.	2	38.9	5.7	1.04794	0.214236	0.051543
4.	2	59	4.9	1.178571	0.126934	0.466325
5.	2	34.2	6.2	1.837776	0.848454	0.085404
